# Supplementary material for: Transcriptional Analysis of a Tripartite Interaction Between Maize (Zea mays, L.) Roots Inoculated with the Pathogenic Fungus Fusarium verticillioides and Its Bacterial Control Agent Bacillus cereus sensu lato Strain B25
Source: Plants (Basel). 2025 Dec 1;14(23):3661. doi: 10.3390/plants14233661 (PMC12693999; doi:10.3390/plants14233661)
Supplement: Supplementary file 1 [file plants-14-03661-s001.zip › plants-3983970-Supplementary Figures.pdf]

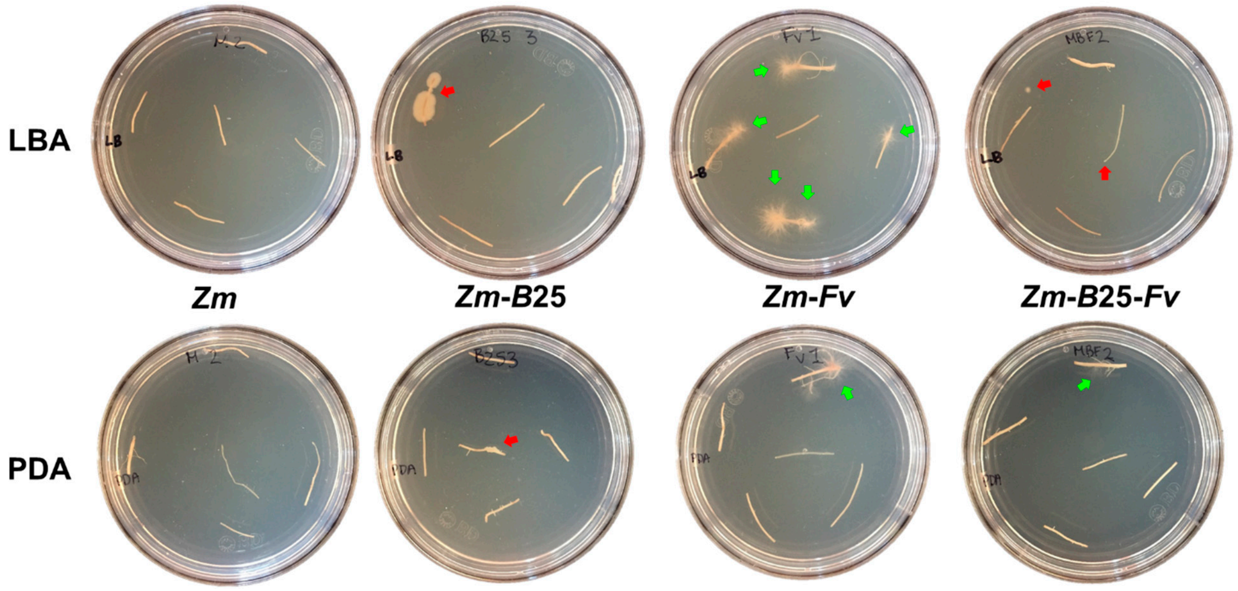

Supplementary Figure S1. Superficially disinfected maize root pieces showing the growth of *B*25 (red arrows) or *Fv* (green arrows) at 7 dpi, 48 h after incubation on media. LBA= Luria Bertani agar. PDA= Potato dextrose agar.

https://doi.org/10.3390/xxxxx

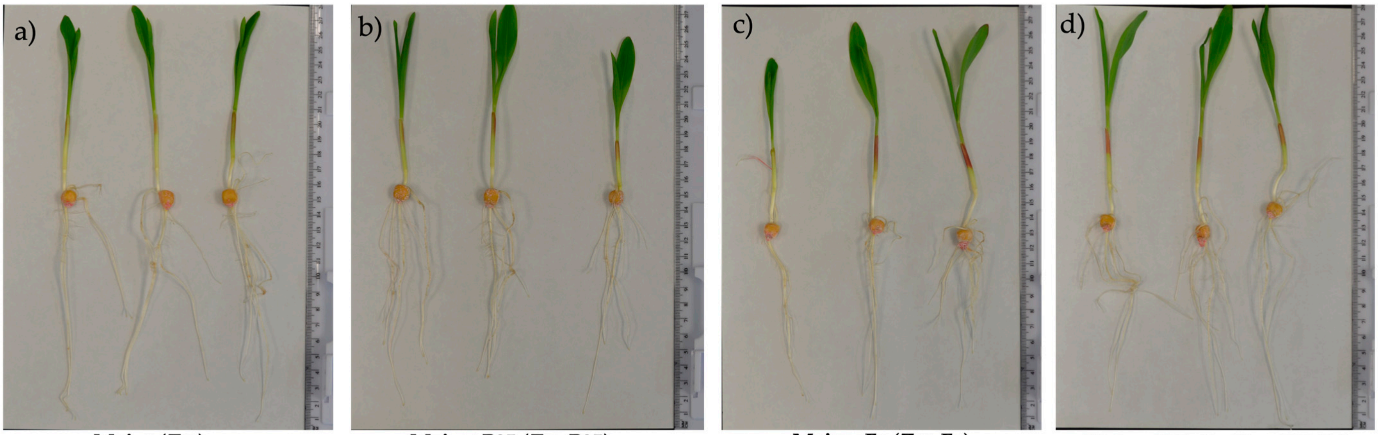

Maize (*Zm*) **Maize+*B*25 (*Zm*-*B*25)** **Maize+*Fv* (*Zm*-*Fv*)** **Maíze+*B*25+*Fv* (Zm-*B*25-*Fv*)**

Supplementary Figure S2. Maize seedlings at 7 dpi at the time of harvest for root RNA extraction. a) control non-inoculated seedlings, b) maize inoculated with *B*25, c) maize inoculated with *Fv*, d) maize inoculated with both *B*25 and *Fv*.

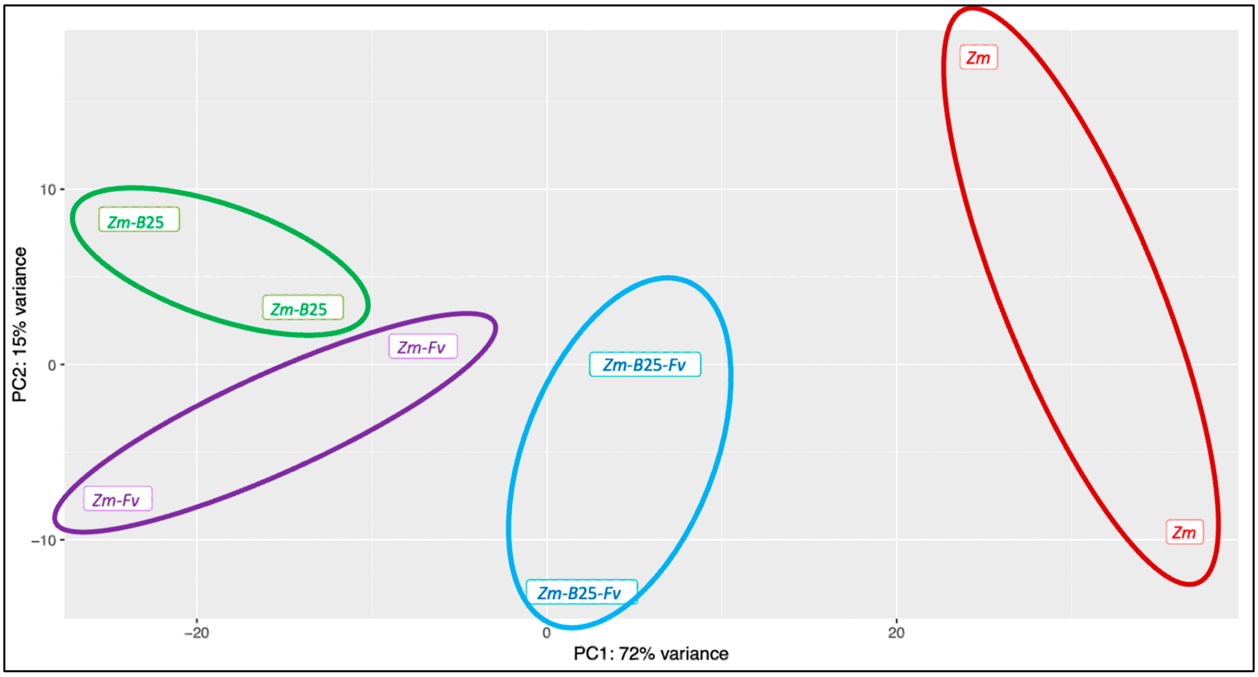

Supplementary Figure S3. Principal component analysis of maize root transcriptional profiles using normalized expression values.
