# Supplementary material for: Transcriptional Analysis of a Tripartite Interaction Between Maize (Zea mays, L.) Roots Inoculated with the Pathogenic Fungus Fusarium verticillioides and Its Bacterial Control Agent Bacillus cereus sensu lato Strain B25
Source: Plants (Basel). 2025 Dec 1;14(23):3661. doi: 10.3390/plants14233661 (PMC12693999; doi:10.3390/plants14233661)
Supplement: Supplementary file 1 [file plants-14-03661-s001.zip › Supplementary Methods.pdf]

plants

MDPI

Supplementary Methods

Enhanced Functional Annotation of RNA-Seq Data: Integrating B73 RefGen_v4 and RefGen_v5

Clean RNA-Seq reads were mapped to the B73 RefGen_v5 maize genome [1]. Since gene function annotations are useful for hypothesis generation, DEGs' biological identity was assessed by collecting information regarding chromosome location, as well as functional and architectural annotation. Given that gene annotation of the B73 RefGen v5 (Zm00001eb) is not as robust as the B73 RefGen v4 (Zm00001d), we combined both by taking advantage of the association between v4 and v5 gene models (https://download.maizegdb.org/Pan-genes/B73_gene_xref/B73v5_to_B73v4.tsv). Gene description was completed with information from Phytozome v13 (https://phytozome-next.jgi.doe.gov/info/Zmays_RefGen_V4), whereas functional and architectural annotation included gene ontology (GO) for gene function prediction [2,3], PFAM for protein domains [4], and InterPro for protein signatures [5]. Importantly, putative orthologous genes in *Arabidopsis thaliana* (*Ath*) and *Oryza sativa* (*Osa*) corresponding to the identified DEGs in maize were also included (Supplementary Table 5).

References

1. Portwood II, J.L.; Woodhouse, M.R.; Cannon, E.K.; Gardiner, J.M.; Harper, L.C.; Schaeffer, M.L.; Walsh, J.R.; Sen, T.Z.; Cho, K.T.; Schott, D.A.; et al. MaizeGDB 2018: The Maize Multi-Genome Genetics and Genomics Database. *Nucleic Acids Res* 2019, *47*, D1146–D1154, doi:10.1093/nar/gky1046.

2. Ashburner, M.; Ball, C.A.; Blake, J.A.; Botstein, D.; Butler, H.; Cherry, J.M.; Davis, A.P.; Dolinski, K.; Dwight, S.S.; Eppig, J.T.; et al. Gene Ontology: Tool for the Unification of Biology. *Nat Genet* 2000, *25*, 25–29.

3. Consortium, T.G.O. The Gene Ontology Resource: Enriching a GOld Mine. *Nucleic Acids Res* 2021, *49*, D325–D334, doi:10.1093/nar/gkaa1113.

4. Bateman, A.; Coin, L.; Durbin, R.; Finn, R.D.; Hollich, V.; Griffiths-Jones, S.; Khanna, A.; Marshall, M.; Moxon, S.; Sonnhammer, E.L.L.; et al. The Pfam Protein Families Database. *Nucleic Acids Res* 2004, *32*, D138-41, doi:10.1093/nar/gkh121.

5. Mitchell, A.; Chang, H.-Y.; Daugherty, L.; Fraser, M.; Hunter, S.; Lopez, R.; McAnulla, C.; McMenamin, C.; Nuka, G.; Pesseat, S.; et al. The InterPro Protein Families Database: The Classification Resource after 15 Years. *Nucleic Acids Res* 2015, *43*, D213–D221, doi:10.1093/nar/gku1243.
