# Supplementary material for: Transcriptional Analysis of a Tripartite Interaction Between Maize (Zea mays, L.) Roots Inoculated with the Pathogenic Fungus Fusarium verticillioides and Its Bacterial Control Agent Bacillus cereus sensu lato Strain B25
Source: Plants (Basel). 2025 Dec 1;14(23):3661. doi: 10.3390/plants14233661 (PMC12693999; doi:10.3390/plants14233661)
Supplement: Supplementary file 1 [file plants-14-03661-s001.zip › Supplementary Table 1.pdf]

Supplementary Table 1. Characteristics of the gene-specific primers used to validate the RNA-Seq by RT-qPCR. All primers had an annealing temperature of 60°C.

Condition	ID[a]	Forward	Reverse
Zm -B 25-*Fv*	Zm00001eb375460	ACGCTGTGGTTATGGAGACC	ATTGACGCACACGCTGTTAG
	Zm00001eb229260	ATCCCCAAAGTGTGCAAAAG	CGGGGATGATATTTGGTTTG
	Zm00001eb251380	CGACGAATTTGCTTCAGACA	GTGGTCAAACCCTTTCCAGA
	Zm00001eb029490	CCAACTTCAACGCAGTGAGA	GATACAACCTGCACCGGACT
	Zm00001eb397080	ATCTCGCCACTGCTCAATCT	CCTGGCTCCCATCAGAAATA
Zm -B 25	Zm00001eb124940	TCGCAGAACACAACACACTG	GGTTGCTCTGCTTTGGTATG
Zm -Fv	Zm00001eb041100	CCCGAGTATTCTGCAGTGCT	CCACATCCACAGCATCAGGT
	Zm00001eb419890	GAGCTATGGGACGGGGAGAT	GCAAGGCAATTGTCCAGGAC
	Zm00001eb285030	CAACCAGATCGCCAAGCAC	ACACATAACAGAGAAGACGCACA
	Zm00001eb241870	TTGCATTGTTCAACGCAGCA	CAGCAGAACGGTGAGAAGGG
Housekeeping gene (CDK)	Zm00001eb350890	CCGTCATCGCCTCACGAAGAG	AGAGCCTGCCTTACGGAATTGG

[a] Maize reference identifier (Zea mays B73 v5.0).
