# Supplementary material for: Transcriptional Analysis of a Tripartite Interaction Between Maize (Zea mays, L.) Roots Inoculated with the Pathogenic Fungus Fusarium verticillioides and Its Bacterial Control Agent Bacillus cereus sensu lato Strain B25
Source: Plants (Basel). 2025 Dec 1;14(23):3661. doi: 10.3390/plants14233661 (PMC12693999; doi:10.3390/plants14233661)
Supplement: Supplementary file 1 [file plants-14-03661-s001.zip › Supplementary Table 3.pdf]

**Supplementary Table 3.** Overrepresented GO terms for unique expressed genes from interaction conditions.

| Category | numDEInCat | | | NumInCat | Term | Ontology | adj_pvalue_over_represented | | |
|---|---|---|---|---|---|---|---|---|---|
| | *Zm-B* 25-*Fv* | *Zm-B* 25 | *Zm-Fv* | | | | *Zm-B* 25-*Fv* | *Zm-B* 25 | *Zm-Fv* |
| GO:0030042 | 2 | | | 51 | Actin filament depolymerization | BP | 0.0517 | | |
| GO:0015629 | 3 | | | 52 | Actin cytoskeleton | CC | 0.0061 | | |
| GO:0051015 | 3 | | | 58 | Actin filament binding | MF | 0.0181 | | |
| GO:0008417 | 2 | | | 58 | Fucosyltransferase activity | MF | 0.047 | | |
| GO:0006355 | | 13 | | 98 | Regulation of transcription, DNA-templated | BP | | 0.0094 | |
| GO:0009733 | | 4 | | 98 | Response to auxin | BP | | 0.024 | |
| GO:0043086 | | 3 | | 98 | Negative regulation of catalytic activity | BP | | 0.0421 | |
| GO:0003700 | | 20 | | 128 | Transcription factor activity, sequence-specific DNA binding | MF | | 0.0001 | |
| GO:0003677 | | 24 | | 128 | DNA binding | MF | | 0.0006 | |
| GO:0004857 | | 4 | | 128 | Enzyme inhibitor activity | MF | | 0.0066 | |
| GO:0016705 | | 7 | | 128 | Oxidoreductase activity, acting on paired donors, with incorporation or reduction of | MF | | 0.0079 | |
| GO:0004497 | | 7 | | 128 | Monooxygenase activity | MF | | 0.016 | |
| GO:0005506 | | 7 | | 128 | Iron ion binding | MF | | 0.0348 | |
| GO:0020037 | | 8 | | 128 | Heme binding | MF | | 0.0388 | |
| GO:0016614 | | 2 | | 128 | Oxidoreductase activity, acting on CH-OH group of donors | MF | | 0.0467 | |
| GO:0042549 | | | 2 | 51 | Photosystem II stabilization | BP | | | 0.0175 |
| GO:0044260 | | | 5 | 51 | Cellular macromolecule metabolic process | BP | | | 0.0227 |
| GO:0044238 | | | 5 | 51 | Primary metabolic process | BP | | | 0.0229 |
| GO:0001709 | | | 2 | 51 | Cell fate determination | BP | | | 0.0348 |
| GO:0005634 | | | 24 | 70 | Nucleus | CC | | | 0.0438 |
| GO:0003700 | | | 11 | 64 | Transcription factor activity, sequence-specific DNA binding | MF | | | 0.0024 |
| GO:0003677 | | | 12 | 64 | DNA binding | MF | | | 0.0197 |
| GO:0046872 | | | 11 | 64 | Metal ion binding | MF | | | 0.0468 |

numDEInCat= number of differentially expressed genes in this category.

NumInCat= number of genes in this category.
