# Supplementary material for: Transcriptional Analysis of a Tripartite Interaction Between Maize (Zea mays, L.) Roots Inoculated with the Pathogenic Fungus Fusarium verticillioides and Its Bacterial Control Agent Bacillus cereus sensu lato Strain B25
Source: Plants (Basel). 2025 Dec 1;14(23):3661. doi: 10.3390/plants14233661 (PMC12693999; doi:10.3390/plants14233661)
Supplement: Supplementary file 1 [file plants-14-03661-s001.zip › Supplementary Table 4.pdf]

Supplementary Table 4. GO Enriched Terms from DEGs of interaction conditions.

category	numDEInCat			numInCat	term	ontology	adj_pva
	Zm-B 25-*Fv*	*Zm-B* 25	*Zm-Fv*				*Zm-B* 25-*Fv*
GO:0005576	129	208	210	600	extracellular region	CC	3.45E-33
GO:0140825	41	75	66	138	lactoperoxidase activity	MF	2.21E-13
GO:0020037	112	174	176	607	heme binding	MF	3.03E-20
GO:0042744	42	76	68	158	hydrogen peroxide catabolic process	BP	6.00E-12
GO:0016491	207	331	364	1498	oxidoreductase activity	MF	2.72E-20
GO:0006979	49	85	81	239	response to oxidative stress	BP	2.31E-10
GO:0004601	46	83	78	234	peroxidase activity	MF	3.92E-09
GO:0098869	48	84	81	257	cellular oxidant detoxification	BP	1.23E-08
GO:0009506	40	68	69	189	plasmodesma	CC	1.23E-08
GO:0009505	25	49	46	108	plant-type cell wall	CC	1.56E-05
GO:0046658	36	51	54	131	NA	NA	9.32E-12
GO:0071704	71	99	113	403	organic substance metabolic process	BP	1.81E-10
GO:0016798	53	101	105	429	hydrolase activity, acting on glycosyl bonds	MF	0.00278
GO:0016021	421	731	872	4865	NA	NA	9.35E-07
GO:0016705	64	86	97	360	oxidoreductase activity, acting on paired donors, with incorporation or reduction of molecular oxygen	MF	5.00E-10
GO:0004553	38	77	79	309	hydrolase activity, hydrolyzing O-glycosyl compounds	MF	0.03288
GO:0005618	34	48	54	165	cell wall	CC	6.06E-07
GO:0004497	72	97	110	435	monooxygenase activity	MF	6.16E-10
GO:0035251	30	52	55	177	UDP-glucosyltransferase activity	MF	0.00093
GO:0048046	48	52	71	197	apoplast	CC	1.66E-13
GO:0042221	16	26	29	66	response to chemical	BP	0.00055
GO:0046872	255	428	554	2799	metal ion binding	MF	1.84E-05
GO:0008194	30	52	55	195	UDP-glycosyltransferase activity	MF	0.00435
GO:0003955	8	9	9	11	NAD(P)H dehydrogenase (quinone) activity	MF	1.02E-05
GO:0016747	34	51	56	207	acyltransferase activity, transferring groups other than amino-acyl groups	MF	0.00035
GO:0004364	17	26	31	84	glutathione transferase activity	MF	0.00181
GO:0016762	10	15	14	32	xyloglucan:xyloglucosyl transferase activity	MF	0.00403
GO:0006749	11	18	21	49	glutathione metabolic process	BP	0.01153
GO:0005506	68	93	106	482	iron ion binding	MF	7.00E-07
GO:0006073	10	15	14	34	NA	NA	0.00635
GO:0009699	14	15	19	41	phenylpropanoid biosynthetic process	BP	8.55E-06
GO:0010411	11	16	15	40	xyloglucan metabolic process	BP	0.0052
GO:0006833	12	14	16	37	water transport	BP	0.0003
GO:0015250	12	14	16	39	water channel activity	MF	0.00031
GO:0016709	19	21	24	64	oxidoreductase activity, acting on paired donors, with incorporation or reduction of molecular oxygen, NAD(P)H as one donor, and incorpor	MF	1.84E-05
GO:0009809	8	9	10	16	lignin biosynthetic process	BP	0.00075
GO:0038023	12	16	19	46	signaling receptor activity	MF	0.00487
GO:0015267	13	15	17	46	channel activity	MF	0.00055
GO:0046274	10	10	11	22	lignin catabolic process	BP	0.00025
GO:0052716	10	10	11	22	hydroquinone:oxygen oxidoreductase activity	MF	0.00025
GO:0043295	6	8	9	17	glutathione binding	MF	0.04973
GO:0010427	9	9	12	22	abscisic acid binding	MF	0.00063
GO:0005886	181	288		1741	plasma membrane	CC	7.00E-07
GO:0008171	11	16		40	O-methyltransferase activity	MF	0.00615
GO:0022900	26	33		162	electron transport chain	BP	0.00024
GO:0080043	13	17		50	quercetin 3-O-glucosyltransferase activity	MF	0.00396
GO:0009055	31	47		260	electron transfer activity	MF	0.00831
GO:0035442	5	5		6	dipeptide transmembrane transport	BP	0.00173
GO:0042937	5	5		6	tripeptide transmembrane transporter activity	MF	0.00173
GO:0042938	5	5		6	dipeptide transport	BP	0.00173
GO:0042939	5	5		6	tripeptide transport	BP	0.00173
GO:0071916	5	5		6	dipeptide transmembrane transporter activity	MF	0.00173
GO:0080044	12	16		49	quercetin 7-O-glucosyltransferase activity	MF	0.01153
GO:0015144	11		18	48	carbohydrate transmembrane transporter activity	MF	0.03012
GO:0098542	13		21	63	defense response to other organism	BP	0.01821
GO:0080163	9		11	25	regulation of protein serine/threonine phosphatase activity	BP	0.00213
GO:0003700		251	267	1420	DNA-binding transcription factor activity	MF	
GO:0006355		368	417	2331	regulation of DNA-templated transcription	BP	
GO:0000272		30	28	78	polysaccharide catabolic process	BP	
GO:0043565		129	141	645	sequence-specific DNA binding	MF	
GO:0009653		26	22	79	anatomical structure morphogenesis	BP	
GO:0008152		100	114	498	metabolic process	BP	
GO:0031408		11	13	21	oxylipin biosynthetic process	BP	
GO:0003680		15	15	38	minor groove of adenine-thymine-rich DNA binding	MF	
GO:0034440		8	8	12	lipid oxidation	BP	
GO:0009627		8	9	19	systemic acquired resistance	BP	
GO:0030145	18			91	manganese ion binding	MF	0.00058
GO:0045735	16			74	nutrient reservoir activity	MF	0.00093
GO:0055085	110			1101	transmembrane transport	BP	0.0042
GO:0006952	49			418	defense response	BP	0.00487
GO:0004864	9			31	protein phosphatase inhibitor activity	MF	0.00615
GO:0009753	6			12	response to jasmonic acid	BP	0.00737
GO:0016460	3			3	myosin II complex	CC	0.01793
GO:0010345	4			6	suberin biosynthetic process	BP	0.03631
GO:0000786		30		121	nucleosome	CC	
GO:0005975		122		669	carbohydrate metabolic process	BP	
GO:0030527		26		108	structural constituent of chromatin	MF	
GO:0009664		18		52	plant-type cell wall organization	BP	
GO:0042542		15		39	response to hydrogen peroxide	BP	
GO:0006857		11		21	oligopeptide transport	BP	
GO:0051213		33		138	dioxygenase activity	MF	
GO:0019438		12		29	aromatic compound biosynthetic process	BP	
GO:0016616		37		161	oxidoreductase activity, acting on the CH-OH group of donors, NAD or NADP as acceptor	MF	
GO:0009698		19		60	phenylpropanoid metabolic process	BP	
GO:1990538		18		57	xylan O-acetyltransferase activity	MF	
GO:0016701		4		4	oxidoreductase activity, acting on single donors with incorporation of molecular oxygen	MF	
GO:0042973		13		35	glucan endo-1,3-beta-D-glucosidase activity	MF	
GO:0047498		4		5	calcium-dependent phospholipase A2 activity	MF	
GO:0000981		63		341	DNA-binding transcription factor activity, RNA polymerase II-specific	MF	
GO:0009800		6		10	cinnamic acid biosynthetic process	BP	
GO:0045548		6		10	phenylalanine ammonia-lyase activity	MF	
GO:0000790		22		86	NA	NA	
GO:0016618		5		7	hydroxypyruvate reductase activity	MF	
GO:0017119		21		87	Golgi transport complex	CC	
GO:0009834		17		59	plant-type secondary cell wall biogenesis	BP	
GO:0061630		71		403	ubiquitin protein ligase activity	MF	
GO:0016757		126		764	glycosyltransferase activity	MF	
GO:0016841		7		15	ammonia-lyase activity	MF	
GO:0016763		15		48	pentosyltransferase activity	MF	
GO:0016161		8		18	beta-amylase activity	MF	
GO:0005615		27		123	extracellular space	CC	
GO:0003677			499	2723	DNA binding	MF	
GO:0022857			117	508	transmembrane transporter activity	MF	
GO:0017116			9	15	single-stranded DNA helicase activity	MF	
GO:0006270			14	33	DNA replication initiation	BP	
GO:0045910			10	19	negative regulation of DNA recombination	BP	
