# Supplementary material for: Transcriptional Analysis of a Tripartite Interaction Between Maize (Zea mays, L.) Roots Inoculated with the Pathogenic Fungus Fusarium verticillioides and Its Bacterial Control Agent Bacillus cereus sensu lato Strain B25
Source: Plants (Basel). 2025 Dec 1;14(23):3661. doi: 10.3390/plants14233661 (PMC12693999; doi:10.3390/plants14233661)
Supplement: Supplementary file 1 [file plants-14-03661-s001.zip › Supplementary Table 5.pdf]

Supplementary Table 5. KEGG over-represented Terms of interaction conditions.

KEGG ID	Count			Pop hits	Term name	adj_pvalue_over_represented		
	Zm-B* 25-*Fv	***Zm-B* 25**	***Zm-Fv***			***Zm-B* 25-*Fv***	***Zm-B* 25**	***Zm-Fv***
zma00940	57	101	92	205	Phenylpropanoid biosynthesis	1.16E-24	1.81E-47	6.27E-27
zma01110	152	286	316	1615	Biosynthesis of secondary metabolites	4.92E-14	5.76E-31	3.04E-14
zma01100	218	403	479	2991	Metabolic pathways	1.34E-09	2.61E-20	3.86E-07
zma00941	14	22	22	47	Flavonoid biosynthesis	2.79E-06	2.53E-09	1.08E-06
zma00073	12	11	13	34	Cutin, suberine and wax biosynthesis	2.93E-06	0.0021	0.0031
zma00402	7	8	7	13	Benzoxazinoid biosynthesis	8.31E-05	0.0002	0.009
zma00480	19	28	35	121	Glutathione metabolism	0.0002	8.81E-05	7.85E-05
zma00999	14	20	18	74	Biosynthesis of various plant secondary metabolites	0.0003	0.0002	0.0275
zma00130	10	18	19	53	Ubiquinone and other terpenoid-quinone biosynthesis	0.0035	2.19E-05	0.0005
zma04075	32	65	71	345	Plant hormone signal transduction	0.0051	7.54E-07	0.0008
zma00591	5	8	8	17	Linoleic acid metabolism	0.0174	0.0014	0.009
zma00052	10	13	17	70	Galactose metabolism	0.0174	0.0445	0.0298
zma00945	6	9	10	31	Stilbenoid, diarylheptanoid and gingerol biosynthesis	0.0316	0.0135	0.0275
zma00905		8		16	Brassinosteroid biosynthesis		0.001	
zma00250		14		67	Alanine, aspartate and glutamate metabolism		0.0176	
zma04016		29		192	MAPK signaling pathway - plant		0.0267	
zma00590		5		13	Arachidonic acid metabolism		0.0434	
zma00944		4		8	Flavone and flavonol biosynthesis		0.0445	
zma04814			30	119	Motor proteins			0.0029
zma03008			31	130	Ribosome biogenesis in eukaryotes			0.0048
zma03030			17	57	DNA replication			0.0065
zma03440			17	62	Homologous recombination			0.0126
zma03410			14	53	Base excision repair			0.0298
zma03450			5	10	Non-homologous end-joining			0.0458
zma00500		30	37	178	Starch and sucrose metabolism		0.0064	0.0127
